# Secondary osteons scale allometrically in mammalian humerus and femur

**DOI:** 10.1098/rsos.170431

**Published:** 2017-11-08

**Authors:** A. A. Felder, C. Phillips, H. Cornish, M. Cooke, J. R. Hutchinson, M. Doube

**Affiliations:** 1 Skeletal Biology Group, Comparative Biomedical Sciences, The Royal Veterinary College, London, UK; 2 Structure and Motion Laboratory, Comparative Biomedical Sciences, The Royal Veterinary College, London, UK; 3 Museums and Archives, The Royal College of Surgeons of England, London, UK

**Keywords:** secondary osteons, intra-cortical bone remodelling, adult body mass, phylogenetic correction, histomorphometry

## Abstract

Intra-cortical bone remodelling is a cell-driven process that replaces existing bone tissue with new bone tissue in the bone cortex, leaving behind histological features called secondary osteons. While the scaling of bone dimensions on a macroscopic scale is well known, less is known about how the spatial dimensions of secondary osteons vary in relation to the adult body size of the species. We measured the cross-sectional area of individual intact secondary osteons and their central Haversian canals in transverse sections from 40 stylopodal bones of 39 mammalian species (body mass 0.3–21 000 kg). Scaling analysis of our data shows that mean osteonal resorption area (negative allometry, exponent 0.23,*R*^2^ 0.54,*p*<0.005) and Haversian canal area (negative allometry, exponent 0.31,*R*^2^ 0.45,*p*<0.005) are significantly related to body mass, independent of phylogeny. This study is the most comprehensive of its kind to date, and allows us to describe overall trends in the scaling behaviour of secondary osteon dimensions, supporting the inference that the osteonal resorption area may be limited by the need to avoid fracture in smaller mammalian species, but the need to maintain osteocyte viability in larger mammalian species.

## 1. Introduction

The cell-driven process of replacing existing cortical bone tissue with new packets of osteoid (a collagenous matrix which later mineralizes, becoming bone) is termed intra-cortical bone remodelling and involves the coordinated action of osteoclasts (bone-resorbing cells) and osteoblasts (osteoid-forming cells) around a central blood vessel, contained within the Haversian canal. Some osteoblasts are buried in the osteoid, becoming osteocytes, but remain connected to each other and to the Haversian canal via the lacunocanalicular network. Secondary osteons, the remnants of such remodelling events, are approximately cylindrical features at a submillimetre scale. In the diaphyses of long bones, osteons are typically oriented in an approximately longitudinal direction [1,2]. They enclose the Haversian canal and are delineated by a collagen-deficient border [3], the cement sheath. In histological sections, the cement sheath is often referred to by its two-dimensional equivalent, the cement line. Haversian canals form a complex vascular network with branching of different types [4].

The function of intra-cortical bone remodelling is the subject of much discussion in the literature. Several mechanical and metabolic functions have been put forward, which are not mutually exclusive. The most widespread hypothesis of bone remodelling function is that bone tissue accumulates micro-damage through everyday loading, and therefore needs to be continuously replaced throughout the lifetime of an organism [5] to avoid fatigue fracture. Although this idea is coherent with micro-damage accumulation in cyclically loaded, synthetic materials, bone remodelling does not seem always to occur in regions of high stress, where the most micro-damage would be expected [6]. This may be because the infilling and mineralization process takes several months to complete (in humans) [7], and thus the presence of large resorption cavities may temporarily weaken highly stressed regions, as well as incurring considerable metabolic costs [6]. Other mechanical functions may be that the cement sheath deflects cracks into less critical directions, thus enhancing toughness by dissipating energy [8] and protecting the nerves, blood vessels and osteocytes within the secondary osteons [9]. The collagen fibre orientation can vary between adjacent lamellae deposited within the resorbed area when the osteon is forming, giving rise to typical osteon ‘morphotypes’ [10,11], which may be adapted to the loading mode [12]. Apart from its roles in maintaining structural integrity, intra-cortical bone remodelling may also regulate calcium homeostasis [13] and mineralization levels [14], process the metabolite flow to ensure ‘intended’ limb proportions during growth [15] or maintain the viability of osteocytes [16]. The loss of osteocyte integrity through overuse or disuse of bone may act as a trigger for intra-cortical remodelling [17], although recent work imaging secondary osteons in the anosteocytic bone of large fish has cast some doubt upon this hypothesis [18].

In mammals, secondary osteons are typically found only in species of adult mass greater than 2 *kg*, although they can be induced by supra-physiological levels of exercise in rats [19], and tend to occur more extensively in larger species [20]. Secondary osteons are also found within the trabeculae of larger species, including some primates [21]. This study, however, focuses on secondary osteons in cortical bone only.

In contrast with the generous attention that scaling relationships of whole bones (e.g. [22–24]) and, more recently, of trabeculae [21,25,26] have received, quantitative measurements of the spatial dimensions of secondary osteons across a large sample of species are yet to be reported in a single study. Furthermore, the presence or absence of a scaling relationship between body mass and secondary osteon dimensions has yet to be fully established. A comparative study of secondary osteon dimensions was performed by Jowsey [27], who, being the first to hypothesize a link between osteon size and species mass, concluded that osteon size increases with body size for species less than 10 *kg*, and then plateaus. The first to address this question with a scaling approach were Mishra & Knothe Tate [28], who, analysing Jowsey’s data, found that osteon and Haversian canal diameters scale with mass *M* as *M*^0.14^ (*R*^2^=0.78) and *M*^0.21^ (*R*^2^=0.75), respectively. Mishra [29] later added data from Tarach & Czaja [30] to this scaling analysis, correcting the previous estimates of scaling relationships for osteon and Haversian canal diameter with body mass to *M*^0.12^ (*R*^2^=0.73) and *M*^0.17^ (*R*^2^=0.67), respectively. These studies [28,29] remain the only scaling studies of osteon size we are aware of.

Previously established scaling exponent estimates of osteon size [28,29] are estimated from linear regression coefficients, which can be problematic when using mass estimates [31]. These estimates are further limited by being based on relatively small sample sizes compared to other scaling studies [21–24]. The evolutionary relationships between species (their phylogeny) mean that data sampled across species cannot be assumed to be independently distributed [32]. Previous scaling analyses of secondary osteon dimensions [28,29] do not account for such phylogenetic effects. The spatial dimensions of secondary osteons and Haversian canals have previously been measured for humans [33] and various other mammalian species (e.g. [34–40]) without considering possible inter-specific scaling effects. Histomorphometrical measurements of secondary osteons remain unreported for most mammalian species. Consequently, a broad overview of scaling of secondary osteon dimensions, able to elucidate overall trends that may be representative of the bio-physical constraints within which the intra-cortical bone remodelling process operates, is still missing.

In this study, we address this shortcoming. We seek to establish the parameters of any scaling relationships between the dimensions of the intra-cortical bone remodelling process and animal size. To achieve this, we first generated composite images of transverse histological sections of mammalian femora and humeri from a museum collection. We then manually digitized the borders of all intact secondary osteons and their Haversian canals in these images using imaging software. Finally, we measured their areas and analysed these measurements using the standardized reduced major axis method [31] and phylogenetically independent contrasts [32].

## 2. Material and methods

### 2.1. Specimens

**Figure 1.**
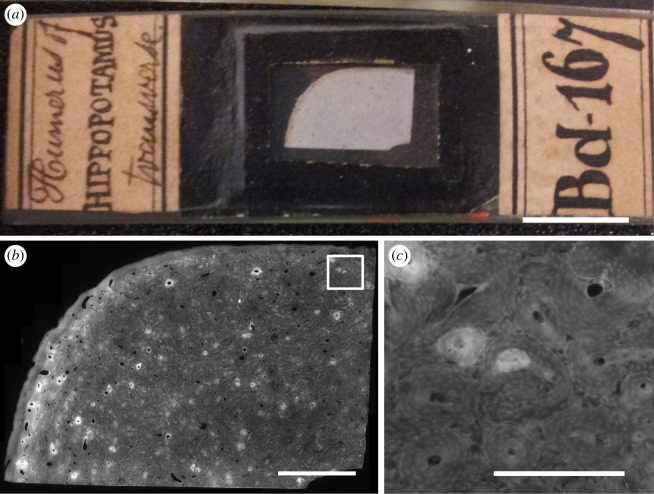
(*a*) Digital photograph of slide Bd 167 from the Quekett collection (scale bar 10 mm). (*b*) Fluorescence microscopic image of the same specimen, automatically stitched from 76 manually captured tile images (scale bar 2.5 mm). (*c*) Inset of (*b*) showing multiple secondary osteons (scale bar 0.5 mm). Images © The Royal College of Surgeons of England, reproduced here with their kind permission.

We obtained access to the historical Bd series of John Thomas Quekett’s collection of microscope slides, which today is part of museum collections at the Royal College of Surgeons (RCS) [41]. Quekett’s collection is one of the earliest museum collections for comparative histology of bone. Sections of larger species covered only part of the whole bone cross section (figure 1). We selected transverse sections from femur or humerus of mammalian species (total 61 specimens, 56 species; see table 1 (humerus) and table 2 (femur) for a list of specimens with searchable identifiers). The rightmost columns specify whether any secondary osteons were found. Our further analyses dealt solely with specimens that had secondary osteons. For each species, we also list an estimate for adult body mass, obtained from the PanTHERIA database [42] for extant species and from the study by Larramendi [43] for fossil proboscideans, which we used as an input for our scaling analyses. We chose stylopodal bones, as we expected the least variation in loading patterns among species (although this variation may still be considerable) and the greatest number of secondary osteons in these bones [20].

**Table 1.** For each humeral specimen: RCS reference number (searchable on the RCS online catalogue SurgiCat), Quekett’s [41] and current terminology, adult body mass estimate [42,43] and whether there were any secondary osteons in it. Secondary literature supporting our assumptions for the terminology can be found in the footnotes.

	common name	binomial used	binomial assumed for		adult body	number of
reference number	used by Quekett	by Quekett	mass estimation	common name	mass (kg)	osteons
RCSMS/Quekett/Division 2/Bd 58	Armadillo	*Dasypus peba*^*a*^	*Dasypus novemcinctus*	Nine-banded armadillo	4	—
RCSMS/Quekett/Division 2/Bd 224	Badger	*Meles meles*	*Meles meles*	European badger	11.9	54
RCSMS/Quekett/Division 2/Bd 91	Smaller species of bat	—^*b*^	*Chiroptera*	Bat sp.	0.1	—
RCSMS/Quekett/Division 2/Bd 113	Dolphin	*Delphinus tursio*^*c*^	*Tursiops truncatus*	Bottle-nosed dolphin	281	12
RCSMS/Quekett/Division 2/Bd 129	Dugong	*Halicore indicus*	*Dugong dugon*	Dugong	295	22
RCSMS/Quekett/Division 2/Bd 6	Echidna	*Echidna hystrix*^*d*^	*Tachyglossus aculeatus*	Short-beaked echidna	4.5	23
RCSMS/Quekett/Division 2/Bd 31	Hare	*Lepus timidus*	*Lepus timidus*	European hare	3.1	16
RCSMS/Quekett/Division 2/Bd 167	Hippopotamus	*Hippopotamus amphibius*	*Hippopotamus amphibius*	Common hippopotamus	1536.3	389
RCSMS/Quekett/Division 2/Bd 266	Lemur	*Lemur tardigradus*^*e*^	*Loris tardigradus*	Red slender loris	0.3	8
RCSMS/Quekett/Division 2/Bd 54	Marmot	*Arctomys marmotta*	*Marmota marmota*	Alpine marmot	4.1	10
RCSMS/Quekett/Division 2/Bd 82	Mole	*Talpa europaea*	*Talpa europaea*	European mole	0.1	—
RCSMS/Quekett/Division 2/Bd 236	Mongoose	*Mangusta Mongos*	*Mungos mungo*	Banded mongoose	1.3	4
RCSMS/Quekett/Division 2/Bd 123	Narwhal	*Monodon monoceros*	*Monodon monoceros*	Narwhal	938.1	7
RCSMS/Quekett/Division 2/Bd 231	Otter	*Lutra vulgaris*	*Lutra lutra*	European otter	8.9	13
RCSMS/Quekett/Division 2/Bd 228	Polecat	—	*Mustela putorius*	European polecat	1	—
RCSMS/Quekett/Division 2/Bd 228	Polecat	—	*Mustela putorius*	European polecat	1	—
RCSMS/Quekett/Division 2/Bd 41	Another species of porcupine	—^*f*^	*Sphiggurus melanurus*	Porcupine sp.	1.9	—
RCSMS/Quekett/Division 2/Bd 117	Porpoise	*Delphinus phocoena*	*Phocoena phocoena*	Harbour porpoise	52.7	—
RCSMS/Quekett/Division 2/Bd 119	Porpoise	*Delphinus phocoena*	*Phocoena phocoena*	Harbour porpoise	52.7	11
RCSMS/Quekett/Division 2/Bd 214	Seal	*Cystophora proboscidea*^*g*^	*Mirounga leonina*	Southern elephant seal	1600	—
RCSMS/Quekett/Division 2/Bd 268	Spider monkey	*Ateles paniscus*	*Ateles paniscus*	Red-faced spider monkey	8.7	7
RCSMS/Quekett/Division 2/Bd 210	Walrus	*Trichechus rosmarus*	*Odobenus rosmarus*	Walrus	1043	1
RCSMS/Quekett/Division 2/Bd 47	Water rat	*Arvicola amphibia*	*Arvicola amphibius*	European water vole	0.1	—
RCSMS/Quekett/Division 2/Bd 17	Wombat	*Phascolomys vombatus*^*h*^	*Vombatus ursinus*	Wombat	26	1

^*a*^See [44].^*b*^Measurement of section cross-sectional area indicates a diameter of approx. 2 mm; We used the average weight of all bat species listed in [45] with diameter 1.5–2.5 mm, i.e. 0.05 kg.^*c*^See [46].^*d*^The genus *Zaglossus* was not discovered until 1876 ([47], as cited in [48]).^*e*^See description in [49].^*f*^Quekett [41] specifies it is not *Hystrix hirsutirostris* (now *H. indica*). To estimate body mass, we scaled weight and mid-diaphyseal diameter data on the African porcupine from [50] isometrically to fit the diameter of the Quekett specimen: For our analysis, we assumed *Sphiggurus melanurus*, which has the appropriate estimated body size, although it is unlikely to be the correct species designation.^*g*^See [51,52].^*h*^See [53].

**Table 2.** For each femoral specimen: RCS reference number (searchable on the RCS online catalogue SurgiCat), Quekett’s [41] and current terminology, adult body mass estimate [42,43] and whether there were any secondary osteons in it. Secondary literature supporting our assumptions for the terminology can be found in the footnotes.

reference	common name	binomial	binomial assumed		adult body	number of
number	used by Quekett	used by Quekett	for mass estimation	common name	mass (kg)	osteons
RCSMS/Quekett/Division 2/Bd 222	Badger	*Meles meles*	*Meles meles*	European badger	11.9	47
RCSMS/Quekett/Division 2/Bd 218	Bear	*Ursus americanus*	*Ursus americanus*	American black bear	110.5	34
RCSMS/Quekett/Division 2/Bd 45	Beaver	*Castor canadensis*	*Castor canadensis*	North American beaver	18.1	—
RCSMS/Quekett/Division 2/Bd 244	Another dog	*Canis familiaris*	*Canis lupus*	Domestic dog	31.8	36
RCSMS/Quekett/Division 2/Bd 137	Fossil elephant from sub-Himalayan tertiary	—^*a*^	*Palaeoloxodon namadicus*	Asian straight-tusked elephant	21000	123
RCSMS/Quekett/Division 2/Bd 272	Entellus monkey	*Semnopithecus entellus*	*Semnopithecus entellus*	Gray langur	12.7	—
RCSMS/Quekett/Division 2/Bd 272	Entellus monkey	*Semnopithecus entellus*	*Semnopithecus entellus*	Gray langur	12.7	—
RCSMS/Quekett/Division 2/Bd 229	Ferret	*Putorius furo var.*	*Mustela putorius*	European polecat	1	—
RCSMS/Quekett/Division 2/Bd 238	Fox	*Canis vulpes*	*Vulpes vulpes*	Red fox	4.8	31
RCSMS/Quekett/Division 2/Bd 264	Flying lemur	*Galeopithecus philippinensis*^*b*^	*Cynocephalus volans*	Philippine flying lemur	1.3	—
RCSMS/Quekett/Division 2/Bd 80	Hedgehog	*Erinaceus europaeus*	*Erinaceus europaeus*	European hedgehog	0.8	—
RCSMS/Quekett/Division 2/Bd 154	Horse	*Equus caballus*	*Equus caballus*	Domestic horse	403.6	9
RCSMS/Quekett/Division 2/Bd 316	Human	*Homo sapiens*	*Homo sapiens*	Human	58.5	160
RCSMS/Quekett/Division 2/Bd 150	Hyrax	*Hyrax capensis*	*Procavia capensis*	Rock hyrax	3	9
RCSMS/Quekett/Division 2/Bd 234	Ichneumon	*Mangusta Ichneumon*	*Herpestes ichneumon*	Egyptian mongoose	3	—
RCSMS/Quekett/Division 2/Bd 8	Kangaroo	*Macropus major*^*c*^	*Macropus giganteus*	Eastern grey kangaroo	33.4	—
RCSMS/Quekett/Division 2/Bd 19	Koala	*Phascolarctos fuscus*	*Phascolarctos cinereus*	Koala	6.5	77
RCSMS/Quekett/Division 2/Bd 270	Short-tailed monkey	*Macacus rhesus*	*Macaca mulatta*	Rhesus macaque	6.5	70
RCSMS/Quekett/Division 2/Bd 63	Manis	*Manis pentadactyla*	*Manis pentadactyla*	Chinese pangolin	4.7	16
RCSMS/Quekett/Division 2/Bd 139	—	*Mastodon*^*d*^	*Mammut americanum*	American mastodon	7800	133
RCSMS/Quekett/Division 2/Bd 52	Mouse	*Mus musculus*	*Mus musculus*	House mouse	0	—
RCSMS/Quekett/Division 2/Bd 179	Napu Musk-deer	*Moschus napu*	*Tragulus napu*	Greater mouse-deer	5.3	—
RCSMS/Quekett/Division 2/Bd 195	Nylghau	*Nylghau*	*Boselaphus tragocamelus*	Nilgai antelope	182.3	87
RCSMS/Quekett/Division 2/Bd 25	Opossum	*Didelphis virginiana*	*Didelphis virginiana*	Virginia opossum	2.4	—
RCSMS/Quekett/Division 2/Bd 276	Orang-utan	*Pithecus satyrus*	*Pongo pygmaeus*	Orangutan sp.	53.4	101
RCSMS/Quekett/Division 2/Bd 232	Otter	*Lutra vulgaris*	*Lutra lutra*	European otter	8.9	—
RCSMS/Quekett/Division 2/Bd 203	Ox	*Bos taurus*	*Bos taurus*	Domestic cattle	618.6	15
RCSMS/Quekett/Division 2/Bd 37	Paca	*Coelogenys Paca*	*Cuniculus paca*	Paca	8.2	11
RCSMS/Quekett/Division 2/Bd 163	Pig	*Sus scrofa*	*Sus scrofa*	Domestic pig	84.5	24
RCSMS/Quekett/Division 2/Bd 258	Puma	*Felis concolor*	*Puma concolor*	Puma	54	119
RCSMS/Quekett/Division 2/Bd 33	Rabbit	*Lepus cuniculus*	*Oryctolagus cuniculus*	European rabbit	1.6	2
RCSMS/Quekett/Division 2/Bd 220	Raccoon	*Procyon lotor*	*Procyon lotor*	Raccoon	6.4	14
RCSMS/Quekett/Division 2/Bd 49	Rat	*Mus rattus*	*Rattus rattus*	Black rat	0.1	—
RCSMS/Quekett/Division 2/Bd 185	Rein-Deer	*Cervus tarandus*	*Rangifer tarandus*	Reindeer	109	61
RCSMS/Quekett/Division 2/Bd 199	Sheep	*Ovis Aries*	*Ovis aries*	Domestic sheep	39.1	72
RCSMS/Quekett/Division 2/Bd 67	Three-toed sloth	*Bradypus tridactylus*	*Bradypus tridactylus*	Three-toed sloth	4.4	19
RCSMS/Quekett/Division 2/Bd 255	Tiger	*Felis tigris*	*Panthera tigris*	Tiger sp.	161.9	222
RCSMS/Quekett/Division 2/Bd 65	Two-toed sloth	*Choloepus didactylus*	*Choloepus didactylus*	Two-toed sloth	6.7	24

^*a*^*Palaeoloxodon namadicus* femora were frequently found before 1855 in the ‘sub-Himalaya’ [54], alongside remains from several other extinct proboscidean species. Being an elephantid [55], *P. namadicus* fits the description ‘fossil elephant’ well, although we cannot exclude *Elephas hysudricus*.^*b*^See [56].^*c*^See [57].^*d*^Some Asian and European species of *Mammut* also existed. The literature on this was unfortunately too vast for the scope of this study.

In some cases, the species was unclear, due to missing or antiquated binomial or common names in the historical documentation of the slides [41]. When this occurred, we usually deduced the taxonomy from the description in the catalogue entry. For some species, we additionally resorted to secondary literature (See footnotes of tables 1 and 2) to establish a species with more confidence. Out of the six more markedly uncertain cases, two (Bd 137, ‘fossil elephant’ and Bd 139, *Mastodon* sp.) had secondary osteons and were therefore relevant to our main analysis. Analysis of these specimens, as they were fossils, was additionally complicated by possible diagenetic effects. However, scaling parameter estimates calculated excluding these specimens remained comfortably within the overall confidence intervals throughout. Thus, excluding these specimens would not have altered our conclusions and we included them for their comparative value.

### 2.2. Historical details on specimen origin and preparation

The specimens we imaged are likely to have been prepared in different ways by several people. Some display commercial coverslip paper of the time and may have been purchased by Quekett, while others are likely to have been produced in-house by Quekett or his collaborators. In his treatise on the use of the microscope, Quekett describes dry and wet preparation methods for thin sections of bone, and examples of both are present in the collection today [58]. Exact anatomical location of our samples is unknown, although most seem to be taken along the diaphysis, based on seeing smooth endosteal surfaces with no trabeculae, except, as expected, in the two sloth species [59] and some of the marine mammals [60]. Specimens from larger species consisted of only partial cross sections (e.g. figure 1*a*). Therefore, circumferential position was also unknown.

The origin of the samples used in this study is unclear. Some are likely to have originated from the Zoological Society of London (ZSL), London Zoo, as Richard Owen, for whom Quekett was initially an assistant before becoming his successor, had the right to claim any freshly deceased animal there. Others may have come from skeletons donated to the RCS by John Gould and other nineteenth-century travelling naturalists. Such donations are described by Quekett in his diaries (e.g. entries of 1 October 1840 and 1 December 1842, accessed at RCS library (MS0027/1)), and sections may have been prepared for the microscope later. Another possible source of specimens is the ZSL Osteological Museum, suggested by a letter from 1840 by ZSL honorary secretary William Ogilby offering this collection to the Hunterian Museum (RCS-MUS/11/1/14, item 52).

### 2.3. Imaging

We imaged the specimens in reflected light fluorescence microscopy using a Leitz Laborlux 12 microscope with a built-in LED light (Excitation 450–490 nm). We manually took overlapping 16-bit grey-scale images, each 1024 by 1024 pixels, covering the entire specimen (4× magnification, 0.12 numerical aperture, pixel size 1.64 μ*m*) with an Orca FLASH4.0LT C11440 digital camera (Hamamatsu Photonics, Hamamatsu City, Japan) and F56-L019 filterset (AHF Analysentechnik, Tübingen, Germany). Consequently, the contrast in the images was generated by green autofluorescence induced by a blue light. In total, we took 3416 images, resulting in an average of 58 image tiles/specimen (minimum 6 (specimen number Bd 91), maximum 180 (Bd 123)). We composed images of entire specimens by applying the Grid/Collections stitching plug-in ([61], v. 1.2) of the open-source image processing software Fiji ([62], v. 1.51) to the image tiles.

**Figure 2.**
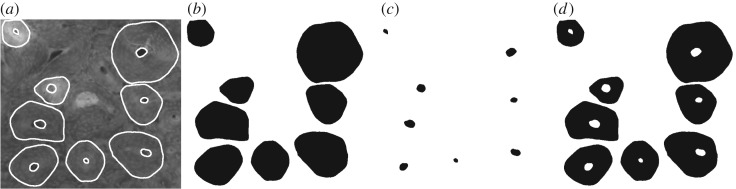
Visualization of measured data. (*a*) Close-up of some of the intact secondary osteons and their Haversian canals from a hippopotamus humerus (Bd 167), traced in white (image © The Royal College of Surgeons of England, reproduced here by their kind permission) (image width: 1.03 mm). (*b*) Binary file created to measure the osteon areas. (*c*) Binary file created to measure the canal areas. (*d*) Binary file created by subtracting the previous binary files from each other to measure the infill areas.

We found intact secondary osteons in 40 specimens (14 humeri, 26 femora) from 39 species. Using a Wacom digitizer (Wacom, Saitama, Japan), we manually traced the boundary of intact secondary osteons and their Haversian canals in the images. To identify secondary osteons, we followed a method used in previous studies of osteonal dimensions [14,33]. Specifically, this involved classifying a feature as an intact secondary osteon if more than 90% of the cement line and the entire canal boundary were visible, and the aspect ratio (maximum diameter/minimum diameter) was less than 2, to prevent atypical osteon variants from confounding our area measurements. For the same reason, drifting osteons [63] were included if we could draw a trace around the osteon from the non-drifting side of the cement line along a clear lamellar boundary, and excluded otherwise, as previously done in [14]. We found a fracture callus in specimen Bd 270, which we excluded from analysis. We stored the osteon area (the area within the cement line, which is the cross section of the cement sheath), the canal area (the area enclosing the Haversian canal) and the infill area (the difference between osteon area and canal area) for each osteon using a custom ImageJ macro (figure 2). From these directly measured quantities, we also computed two derived quantities for each intact secondary osteon: the infill distance (the distance between the canal border and the cement line, assuming perfectly circular osteons of equivalent area with concentric Haversian canals of equivalent area), and the infill ratio (infill area divided by its corresponding osteon area) using custom ImageJ macros.

The number of secondary osteons present in each specimen varied from 1 to 389 (last columns in tables 1 and 2). To prevent our results from being biased by specimens with low numbers of osteons (often small species, some artiodactyls or marine mammals), we repeated our analysis excluding specimens with fewer than 10 measured osteons. This offered some additional robustness to our analyses, because any false positives (features wrongly classified as secondary) would have a particularly high weight in specimens with few osteons. Again, the results for scaling exponents and elevations were within the bounds obtained including all specimens.

### 2.4. Statistical analyses

Scaling analyses aim to describe the relationship between a quantity of interest and body size, usually mass. The quantities of interest for this study included the mean, minimum and maximum of the direct (osteon area, canal area, infill area) and derived (infill ratio, infill distance) measurements described in the previous subsection. As our quantities of interest could not always be assumed to be normally distributed, we distinguished between cases where this assumption was justified and cases where it was not. This was assessed using a Shapiro–Wilk test (with *p*<0.05) in R ([64], v. 3.3.2). We only explicitly state cases where the assumption of normality was unjustified in the results.

To describe the statistical significance of the association between any of our variables of interest and body mass, we report *p*-values. In all our analyses, we used *p*<0.005 and *p*<0.05 as cut-off values for high and mild statistical significance, respectively.

To describe the strength of association between any of our variables of interest and body mass, we reported squared correlation coefficients. The squared correlation coefficient was Pearson’s *R*^2^ if the data could be assumed to be normal, and the Spearman’s *ρ*^2^ otherwise. We interpreted significant relationships with squared correlation coefficients greater than 0.3 as strong, and otherwise as weak.

In cases where the data were not normally distributed (infill ratio), we report the median instead of the mean as a measure of central tendency. In cases where the data were normally distributed, but the fact that osteons which had not finished infilling may have biased our measurements (canal area, infill area and infill distance), we additionally report the median as a measure of central tendency that is robust to outliers.

We assumed our quantities of interest *Y* obeyed a power law *Y* =*aM*^*b*^, where *M* denotes body mass, *b* the scaling exponent and *a* the elevation. We obtained estimates, lower and upper bounds for both the scaling exponent *b* (i.e. the slope on a logarithmic plot) and the elevation *a* using the standardized (reduced) major axis from the R package SMATR 3 [65] on the base-10 logarithms of our variables of interest. The standardized major axis is the preferred regression method for scaling studies, as it takes into account measurement errors of differing variance on the *x*- and *y*-axes [31], and allows testing of hypothesized slopes. We used the same software to test for the null hypotheses of isometry and no correlation. Isometry entails proportional increase with body mass [66], and therefore slope $b=\frac{1}{3}$ for the infill distance, *b*=0 for the infill ratio and $b=\frac{2}{3}$ for all other (area) measurements. Additionally, in order to control for phylogenetic relationships of varying degree between species, we calculated phylogenetically independent contrasts [32] using the pic function of the R package ape [67] and a species-level phylogenetic tree, including branch lengths [68]. Entries for the extinct proboscideans in our sample were added with a custom R script, using estimated branch lengths from a previous study[69]. SMATR was used to estimate the scaling exponent of independent contrasts, while enforcing zero elevation, as justified in [31]. The rationale behind using phylogenetically independent contrasts is that if the scaling relationships of the contrasts differ strongly from the ordinary scaling relationship for a quantity of interest, phylogeny is an important factor in the study and the data are not as independent as otherwise assumed.

If scaling exponent and elevation estimates calculated for femora and humeri individually were not comfortably within 95% CI of the estimates of combined stylopod data, we report the data separately. Otherwise, we pooled the data for analysis.

Our R code [70] and ImageJ macros [71] are available online.

## 3. Results

### 3.1. Direct measurements

**Figure 3.**
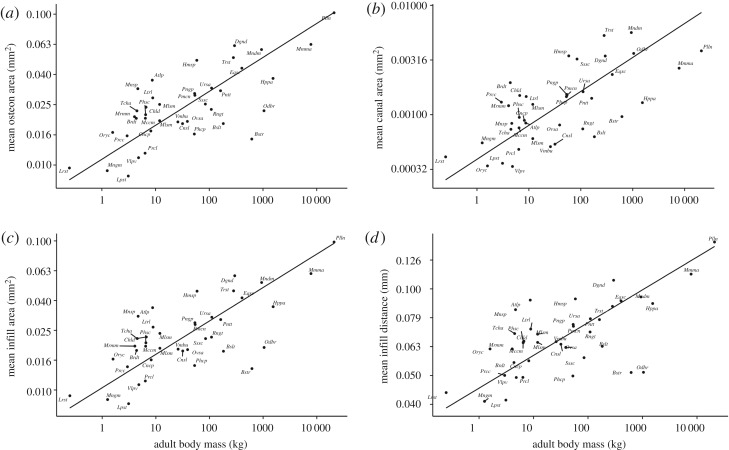
Log–log plots of per species means of the secondary osteon cross-sectional parameters and estimated body mass, where we found a significant relationship (*R*^2^>0.3, *p*<0.005). Estimates, sample square correlation coefficients and *p*-values for all parameters we measured can be found in table 3 and 4.

**Figure 4.**
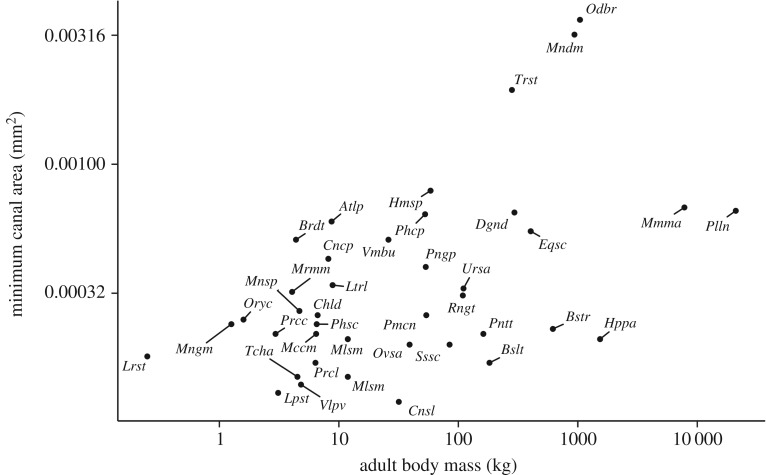
Log–log plot of per species minima of canal area and estimated body mass. Body mass relates to less than 30% of variation in the minimum osteon area, canal area and infill area (table 3), indicating that ‘narrow’ secondary osteons are found throughout mammalian species.

Mean osteon area, canal area and infill area showed negative allometric (i.e. less than isometric) scaling (*b*=0.23, *b*=0.31 and *b*=0.22, respectively), as visualized in figure 3. These relationships were different to both null hypotheses (scaling-invariance and isometry) with high significance (*p*<0.005). Sample squared Pearson’s correlation coefficients (*R*^2^) showed that our fits for the means of directly measured variables, where highly significant, explained between 45% and 52% of the variation in our data. The maxima and medians for these quantities showed the same gross scaling pattern (scaling exponents within 0.02 of each other) as the means, with similar values for *p* and *R*^2^. The maximum canal area scaled close to isometry, but was still negatively allometric with mild significance (*p*<0.05). The variation in our data for minimum areas was considerably less well explained by our fits. The correlation of minimum osteon area and minimum infill area with body mass was weak (*R*^2^=0.14 and *R*^2^=0.12, respectively, *p*<0.05), and was lost completely when excluding samples that had fewer than 10 osteons (*p*=0.37). The minimum canal area was not normally distributed (*p*<0.005, Shapiro–Wilk), and a Spearman’s rank correlation test with body mass yielded a similarly weak relationship (*ρ*^2^=0.21,*p*<0.005; figure 4). The elevation for the mean canal area was 390 μ*m*^2^, two orders of magnitude less than its analogues for osteon area and infill area, which corresponds to a mean canal diameter of 11.1 μ*m* (assuming circular canals). Estimates and 95% CI for *a* and *b* of directly measured quantities are summarized in table 3.

**Table 3.** Summary of the scaling analysis for the osteon area, canal area and infill area: slope estimate with lower and upper bounds (*b*,*b*^−^,*b*^+^), elevation estimate with lower and upper bounds (*a*,*a*^−^,*a*^+^), sample squared correlation coefficient (*R*^2^), and *p*-values for no correlation (*p*_*uncorrelated*_) and isometric scaling (*p*_*isometry*_) null.

	osteon area			
	mean	minimum	maximum	
*b*	0.23	0.23	0.27	
*b*^−^	0.18	0.17	0.21	
*b*^+^	0.28	0.31	0.33	
*a*	0.011	0.0047	0.018	
*a*^−^	0.0087	0.0034	0.014	
*a*^+^	0.014	0.0065	0.024	
*R*^2^	0.54	0.14	0.53	
*p*_*uncorrelated*_	<0.005	0.017	<0.005	
*p*_*isometry*_	<0.005	<0.005	<0.005	
		canal area		
	mean	minimum	maximum	median
*b*	0.31	0.32	0.46	0.3
*b*^−^	0.24	0.25	0.36	0.23
*b*^+^	0.39	0.43	0.58	0.37
*a*	0.00039	0.00011	0.00077	0.00031
*a*^−^	0.00028	0.000071	0.00047	0.00023
*a*^+^	0.00055	0.00016	0.0013	0.00043
*R*^2^	0.45	0.27	0.46	0.47
*p*_*uncorrelated*_	<0.005	<0.005	<0.005	<0.005
*p*_*isometry*_	<0.005	<0.005	<0.005	<0.005
		infill area		
	mean	minimum	maximum	median
*b*	0.22	0.22	0.27	0.24
*b*^−^	0.18	0.17	0.21	0.19
*b*^+^	0.28	0.3	0.34	0.3
a	0.01	0.0044	0.017	0.0091
*a*^−^	0.0083	0.0032	0.013	0.0071
*a*^+^	0.013	0.0061	0.023	0.012
*R*^2^	0.52	0.12	0.5	0.47
*p*_*uncorrelated*_	<0.005	0.03	<0.005	<0.005
*p*_*isometry*_	<0.005	<0.005	<0.005	<0.005

### 3.2. Derived measurements

**Figure 5.**
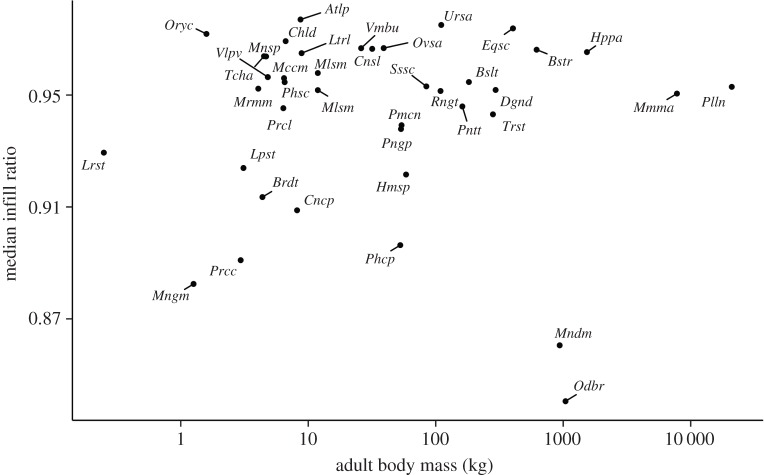
Log–log plot of per species median infill ratio and estimated body mass. Infill ratio does not scale with body mass, and is kept greater than 85% throughout mammalian species.

Shapiro–Wilk tests indicated that the logarithms of the mean, maximum and median infill ratio were not normally distributed (*p*<0.005). We therefore performed non-parametric (Spearman’s rank) correlation tests on these data, which showed that the mean, median and maximum infill ratio were not correlated with animal body mass (*p*>0.18 in all cases). The minimum infill ratio was not normally distributed (*p*<0.005, Shapiro–Wilk test), and a non-parametric test showed a very weak correlation with body mass (*p*<0.05,*ρ*^2^=0.12). The significance of this correlation was lost when analysing the data within separate groups of femoral and humeral specimens. The median infill ratio ranged from 84% to 99% (figure 5).

The mean infill distance scaled with negative allometry (*b*=0.11,*R*^2^=0.44,*p*<0.005), indicating that while the infill distance increases on average with species size in absolute terms, larger mammalian species have a relatively smaller mean infill distance (table 4). We observed similar relationships for the median and the maximum infill distance. However, the minimum infill distance is independent of species size, possibly due to the fact we included osteons at an early infilling stage. The overall maximum infill distance (found in specimen Bd 137, straight-tusked elephant) was 180 μ*m*.

**Table 4.** Summary of the scaling analysis for the infill ratio and the infill distance: slope estimate with lower and upper bounds (*b*,*b*^−^,*b*^+^), elevation estimate with lower and upper bounds (*a*,*a*^−^,*a*^+^); for the infill distance, sample squared correlation coefficient, and *p*-values for no correlation (*p*_*uncorrelated*_) and isometric scaling (*p*_*isometry*_) null hypotheses are reported. We report the median instead of the mean, and Spearman’s *ρ*^2^, for the infill ratio, as the data were not normally distributed, as indicated by the *p*-values (*p*_*shapiro*–*wilk*_) of normality tests. Isometry was not tested for the infill ratio, as it did not scale in the first place.

	infill ratio			
	median	minimum	maximum	
*b*	−0.015	−0.022	−0.019	
*b*^−^	−0.02	−0.029	−0.026	
*b*^+^	−0.011	−0.016	−0.014	
*a*	1	0.99	1	
*a*^−^	0.97	0.96	0.99	
*a*^+^	1	1	1	
*p*_*shapiro*–*wilk*_	<*0*.*005*	<*0*.*005*	<*0*.*005*	
*ρ*^2^	0.0022	0.12	0.02	
*p*_*non*-*param*._	0.77	0.029	0.38	
		infill distance		
	mean	minimum	maximum	median
*b*	0.11	0.11	0.13	0.12
*b*^−^	0.089	0.084	0.097	0.093
*b*^+^	0.14	0.16	0.17	0.15
*a*	0.045	0.031	0.053	0.044
*a*^−^	0.04	0.027	0.046	0.038
*a*^+^	0.051	0.037	0.062	0.05
*R*^2^	0.44	0.073	0.33	0.39
*p*_*uncorrelated*_	<*0*.*005*	0.091	<*0*.*005*	<*0*.*005*
*p*_*isometry*_	<*0*.*005*	<*0*.*005*	<*0*.*005*	<*0*.*005*

These results are summarized in table  4.

### 3.3. Phylogenetic signal

Scaling exponent estimates for phylogenetically independent contrasts of all variables of interest were slightly higher, but in all cases within the 95% CI obtained without any correction for phylogeny (table 5), indicating minimal effect of phylogeny on the scaling relationships.

**Table 5.** Slope estimates, *R*^2^, and *p*-values for scale-invariant (*p*_*uncorrelated*_) and isometric scaling (*p*_*isometry*_) null hypotheses on the independent contrasts for our data.

	*b*_*independent* *contrasts*_	*p*_*uncorrelated*_	*R*^2^	*p*_*isometry*_
mean osteon area	0.265	<0.005	0.474	<0.005
minimum osteon area	0.291	0.015	0.159	<0.005
maximum osteon area	0.277	<0.005	0.373	<0.005
mean canal area	0.333	<0.005	0.439	<0.005
minimum canal area	0.368	0.005	0.204	<0.005
maximum canal area	0.500	<0.005	0.323	<0.005
mean infill area	0.265	<0.005	0.456	<0.005
minimum infill area	0.286	0.020	0.145	<0.005
maximum infill area	0.283	<0.005	0.340	<0.005
mean infill ratio	−0.015	0.165	0.054	<0.005
minimum infill ratio	−0.023	0.049	0.106	<0.005
maximum infill ratio	−0.024	0.341	0.026	<0.005
mean infill distance	0.129	<0.005	0.369	<0.005
minimum infill distance	0.153	0.068	0.092	<0.005
maximum infill distance	0.142	0.009	0.180	<0.005

## 4. Discussion

**Figure 6.**
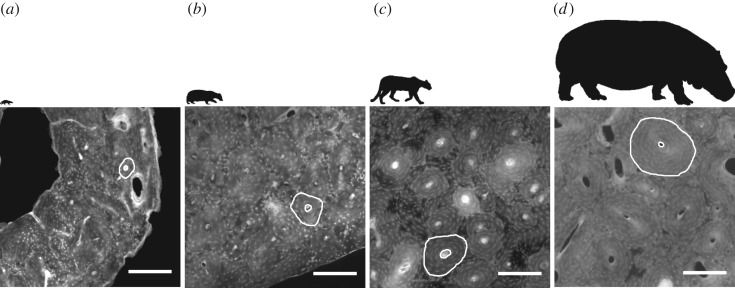
Close-ups of the bone cortex in four specimens with silhouettes reflecting the size differences above. (*a*) Banded mongoose, (*b*) European badger, (*c*) puma, (*d*) common hippopotamus (all scale bars 0.25 mm). Images © The Royal College of Surgeons, and reproduced here with their kind permission.

Our analysis shows that cross-sectional parameters of secondary osteons and their Haversian canals scale with negative allometry in the mammalian humerus and femur. Therefore, our key finding is that, where intra-cortical remodelling is present, larger mammals have larger osteonal and Haversian canal areas compared to smaller mammalian species in absolute terms, but smaller osteonal and Haversian canal areas in relative terms (figure 6 and table 3). The distance between the canal border and the cement line also showed a negatively allometric relationship with animal size, whereas the ratio of the infill area to the osteon area does not scale (table 4). The range of species, clades and adult body mass considered here comfortably exceeds the only previous scaling study concerning itself with secondary osteons [28]. In general, our data support the accepted view that extensive remodelling [20] is present only in larger species. We did not find any secondary osteons in some medium-sized terrestrial species (Bd 8, *Macropus giganteus*, 33 kg; Bd 45, *Castor canadensis*, 18 kg; Bd 272, *Semnopithecus entellus*, 12 kg) and a large marine species (Bd 214, *Mirounga leonina*, 1600 kg), which might be due to the anatomical location or age of the specimen sampled.

### 4.1. Physiological and mechanical constraints on osteon dimensions

Previous studies showed a tight coupling between bone resorption and formation in intra-cortical bone remodelling. Qiu *et al*. [72] demonstrated a strong (*r*=0.97) quadratic relationship between osteon perimeter and infill area in young and elderly humans. Another study [73] showed strong correlations (*r*>0.95) between osteon area and infill area for osteons of varying sizes from several species. The size invariance of the infill ratio, and similar exponent estimates for the osteon area and the infill area (differing by less than 0.01) reported here extend this finding to an unprecedented range of mammalian species with secondary osteons. Thus, seemingly, a similar percentage of resorbed bone is filled back in, independent of body size, suggesting that it is crucial for all mammalian limb bones to keep their overall porosity at a similar level, because porosity is an important determinant of bone strength [74].

Sample squared correlation coefficients are lower for the minimum than for the maximum and mean osteon, canal and infill area. Erythrocyte dimensions may form a lower limit for the cross-sectional area of remote blood vessels such as Haversian canals and do not vary with species size in mammals [75]. Idealizing the minimum canal areas as circles resulted in diameters of approximately 10 μ*m*, which are close to the diameter of an erythrocyte (approx. 8 μ*m*, [76]), supporting this notion. Therefore, occasionally, osteons of large species are narrow relative to the entire, inter-species range we report in the present study (and erythrocytes still fit through the capillaries), but wide osteons do not occur in small species.

On the other hand, upper limits for osteon and canal area may differ for species of varying size: The maximum distance from a blood vessel at which osteocytes remain viable has been reported to be 230 μ*m* [16]. This is close to the maximum infill distance we observed in the larger species of our samples and suggests that maintaining osteocyte viability, combined with the previously mentioned size-invariant infill ratio, constitutes a limiting factor for osteon area in large species. For smaller species, however, this limit is never reached, suggesting that a different mechanism dominates the size of osteons in these animals.

The cortical thickness of the specimen with the smallest estimated body mass and evidence of osteons in our sample (red slender loris, *Loris tardigradus*) ranges between 600 and 850 μ*m*, which is of comparable scale to the estimated diameter of the largest osteon we found overall (580 μ*m*). A resorption area of that size in as thin a cortex is likely to have deleterious consequences for the bone [77]. Creating as large osteons as possible, if an organism can afford the temporary increase in porosity due to a large resorption cavity, may be advantageous from a mechanical point of view after infilling has completed. Larger (and more numerous) osteons have been associated with increased toughness [78–81]. It has more recently been suggested that larger osteons [82] are geometrically more advantageous for resisting bending and compression loads. Studies treating bone as a quasi-brittle material suggest that bone strength and toughness improve with increases in the ratio between dominant inhomogeneity size (i.e. in our case, the typical size of microscopic features such as osteons) and the structural scale (i.e. the bone organ size) [83–85].

Therefore, larger osteons, to the extent to which they do not preclude osteocyte viability, in large species may be explained as tissue-level geometrical adaptations to offset mechanical disadvantages that come with increased size, conceptually similar to adaptations at the organ level described in previous studies such as decreasing bending moments with more upright postures [86], increased limb bone robustness [87], cortical thickness and infilling with trabeculae [88], and adapting number and thickness of trabeculae [21].

It has previously been hypothesized that the energy efficiency of the fluid transport to the outermost osteocytes limits the size of secondary osteons [89]. However, that study based its argument on an idealized osteon model, where the velocity of the fluid flow is constant from lamellar boundary to lamellar boundary. A more recent study, using three-dimensional stacks of electron microscopic images and advanced modelling techniques, has shown a highly heterogeneous flow profile in the canaliculi, casting considerable doubt on this assumption [90]. Furthermore, the upper limit to osteon size of ‘five to six lamellae’, apparently corresponding to 150 μ*m*, proposed in [89] is directly contradicted by observations of up to 13 lamellae in human secondary osteons (e.g. fig. 6 in [91]).

The fact that phylogeny had little effect on our findings is unsurprising, given the diversity of species, clades and ecologies that we sampled from. This further reinforces the notion that our results reflect overall bio-physical limits within which the intra-cortical bone remodelling process operates, and not clade- or species-specific adaptations.

### 4.2. Limitations

The high variability of our data reflects the multitude of species- and specimen-specific characteristics that affect bone remodelling, including age, sex, habitual loading mode, locomotor style, life history, levels of activity, exact specimen body mass, onset of secondary remodelling during ontogeny, environmental influences and more. Specimen age was unknown, but we expect most specimens to be of young adult age, as life expectancy in nineteenth-century zoos was low ([92], as cited in [93]). There is some debate about the relationship between age and osteon size in humans: while most studies support an inverse relationship (narrower osteons in older people) [94,95], some studies show no effect. Osteon size was not correlated with age in macaques [35]. The effect size of age in inter-species comparisons of secondary osteon dimensions (if there is one) is, however, likely to be smaller in magnitude than the relationship of secondary osteons with mass that we show here. Similarly, we could not take sex into account in our analysis, because it was unknown for all our specimens. Habitual loading directions (compression or tension) may affect osteon size, but it is unclear how. Osteons in the artiodactyl and equine calcaneus and the chimpanzee femur have been shown to be narrower in the compression cortex compared to the tension cortex, but the opposite was true of the equine radius and the human femur [12]. Given that it was difficult to establish the anatomical location and orientation from which the samples were taken, we could not control for this fact in this study. This line of thought is, however, promising for future scaling studies including specimens for which the sample location is known, although caution should be exercised when estimating the loading environment from geometrical parameters [96].

The fact that we sampled only complete osteons may have biased some of the data presented here towards secondary osteons that had not finished infilling. This bias may have been alleviated by sampling Haversian canals from secondary osteon fragments, which are more likely to have finished infilling. However, as some of our samples contained only one generation of secondary osteons (and therefore no fragments), this approach would have severely compromised our ability to compare data throughout species. Our method which captures osteons that had not completed infilling (and ones that stopped infilling at an earlier stage than others [97]), gives a more complete picture relevant to the biomechanical situation. Furthermore, median measurements (which are robust to outliers, including those resulting from incompletely infilled osteons) of the canal area, infill area and infill distance displayed essentially the same scaling patterns as the means throughout, so we are confident that this bias did not affect our conclusions.

Because stylopodal dimensions also scale with body size [24], it seems reasonable to speculate that bone size, and not body size, determines osteon dimensions [98]. However, data for calcanei and femora from equine samples, and metapodials and femora from American black bears, suggest that secondary osteon dimensions are similar across different anatomical sites and across bones of varying size within the same species [73]. This makes it appear more likely that osteon dimensions relate to adult body mass, as our data show, and not to bone size. To distinguish the contributions to osteon size of bone organ size and whole body mass in more detail, a further study, similar to ours, comparing osteon dimensions in bones of the same size across species, and in bones of different sizes within species, would be required.

More insights would be gained if we were to measure the secondary remodelling process in three dimensions. However, while we are now able to image resorption cavities effectively using X-ray microtomography [99], for now, the characterization of cement sheaths in three dimensions involves considerable manual effort [4] and could not have been completed within the time scale of this study.

Using a historical collection of slides presented some further limitations. Owing to the large variation in sample cross-sectional area, unknown anatomical location and unclear boundaries of remodelled areas in some specimens, we did not feel comfortable reporting any osteon areal density (osteons *mm*^−2^) measurements, as they would not have been comparable between species. Having only a few (one or two) specimens per species further limits our study, as these specimens may not be representative of the species. This could, for example, have been the case if the animal in question suffered from illnesses affecting bone metabolism or regular locomotor activity. To an extent, knowledge of exact anatomical location, precise taxon as well as specimen age, health and provenance was sacrificed in order to sample a large species and size range. We feel this was important, as we would have been prone to concluding that there was no scaling relationship if we had used a smaller number of different species that are easily accessible, given that (for example) humans have relatively wide, and cattle have relatively narrow, osteons for their size. The use of a historical collection additionally avoided the time, effort and difficulties required to obtain and generate an equivalent collection to a modern standard.

Fluorochrome labelling could have helped validate our results. However, this would have constituted only a partial validation: it would have confirmed whether we characterized the secondary osteons formed during the labelling period correctly (i.e. true positives), but would probably have produced a number of false negatives, namely intact secondary osteons formed prior to fluorochrome injection. Using solely labelled osteons would have reduced the number of osteons sampled, increased the bias towards osteons which had not completed infilling at the time of death or had possibly differing infilling rates, and could have therefore confounded our results. Moreover, obtaining labelled material from a large enough range of species would have posed considerable difficulties.

## 5. Conclusion

In summary, the images of historically important specimens we produced are available (see data accessibility statement below) to researchers for use in future comparative histological studies. The measurements of secondary osteon and Haversian canal area we presented here have the highest number of mammalian species with the most diversity in clades and the broadest range in size to date. Having such a comprehensive sample was crucial for our analysis, but also intrinsically limited by the uncertainty surrounding historical specimens. We performed a scaling analysis of quantities describing the intra-cortical remodelling process across species, which showed negative allometry for osteon, canal and infill area as well as infill distance, while the infill ratio was size-independent. Our results indicate that minimum osteon dimensions correlate only very weakly with animal body mass and might relate to erythrocyte dimensions. By contrast, the upper limits to osteon dimensions display a strong relationship with adult body size: osteons in small species may be restricted to sizes avoiding fatal temporary stress increases around too large resorption cavities, while osteon size in large species may be dictated by the ability to maintain osteocyte viability.

## Data accessibility

Full-resolution images can be obtained from RCS for research purposes upon request (low-resolution versions can be found on the RCS online catalogue SurgiCat by searching for the reference numbers listed in tables 1 and 2). Segmentations of the images, and the raw data of the histomorphometrical measurements are available from the Dryad Digital Repository https://doi.org/10.5061/dryad.637r1 [100]. The code used to obtain our results is available on GitHub [70,71].

## References

[1] Petrtýl M, Heřt J, Fiala P 1996 Spatial organization of the Haversian bone in man. *J. Biomech.* 29, 161–169. (doi:10.1016/0021-9290(94)00035-2) 8849809 10.1016/0021-9290(94)00035-2

[2] Burger EH, Klein-Nulend J, Smit TH 2003 Strain-derived canalicular fluid flow regulates osteoclast activity in a remodelling osteon—a proposal. *J. Biomech.* 36, 1453–1459. (doi:10.1016/S0021-9290(03)00126-X) 14499294 10.1016/s0021-9290(03)00126-x

[3] Davies JE 2007 Bone bonding at natural and biomaterial surfaces. *Biomaterials* 28, 5058–5067. (doi:10.1016/j.biomaterials.2007.07.049) 17697711 10.1016/j.biomaterials.2007.07.049

[4] Maggiano IS, Maggiano CM, Clement JG, Thomas CDL, Carter Y, Cooper DML 2016 Three-dimensional reconstruction of Haversian systems in human cortical bone using synchrotron radiation-based micro-CT: morphology and quantification of branching and transverse connections across age. *J. Anat.* 228, 719–732. (doi:10.1111/joa.12430) 26749084 10.1111/joa.12430 PMC4831337

[5] Martin R 2003 Fatigue damage, remodeling, and the minimization of skeletal weight. *J. Theor. Biol.* 220, 271–276. (doi:10.1006/jtbi.2003.3148) 12602399 10.1006/jtbi.2003.3148

[6] Currey JD, Dean MN, Shahar R 2016 Revisiting the links between bone remodelling and osteocytes: insights from across phyla: what is bone remodelling for? *Biol. Rev.* 92, 1702–1719. (doi:10.1111/brv.12302) 27862887 10.1111/brv.12302

[7] Parfitt AM 1994 Osteonal and hemi-osteonal remodeling: the spatial and temporal framework for signal traffic in adult human bone. *J. Cell. Biochem.* 55, 273–286. (doi:10.1002/jcb.240550303) 7962158 10.1002/jcb.240550303

[8] Koester KJ, Ager JW, Ritchie RO 2008 The true toughness of human cortical bone measured with realistically short cracks. *Nat. Mater.* 7, 672–677. (doi:10.1038/nmat2221) 18587403 10.1038/nmat2221

[9] Martin RB, Burr DB, Sharkey NA, Fyhrie DP 2015 *Skeletal tissue mechanics*, 2nd edn New York, NY: Springer.

[10] Ascenzi A, Bonucci E 1968 The compressive properties of single osteons. *Anat. Rec.* 161, 377–391. (doi:10.1002/ar.1091610309) 4879362 10.1002/ar.1091610309

[11] Bromage TG, Goldman HM, McFarlin SC, Warshaw J, Boyde A, Riggs CM 2003 Circularly polarized light standards for investigations of collagen fiber orientation in bone. *Anat. Rec. B. New. Anat.* 274, 157–168. (doi:10.1002/ar.b.10031) 12964206 10.1002/ar.b.10031

[12] Skedros JG, Keenan KE, Williams TJ, Kiser CJ 2013 Secondary osteon size and collagen/lamellar organization (‘osteon morphotypes’) are not coupled, but potentially adapt independently for local strain mode or magnitude. *J. Struct. Biol.* 181, 95–107. (doi:10.1016/j.jsb.2012.10.013) 23123271 10.1016/j.jsb.2012.10.013

[13] Vajda EG, Kneissel M, Muggenburg B, Miller SC 1999 Increased intracortical bone remodeling during lactation in beagle dogs. *Biol. Reprod.* 61, 1439–1444. (doi:10.1095/biolreprod61.6.1439) 10569987 10.1095/biolreprod61.6.1439

[14] Goldman HM, Hampson NA, Guth JJ, Lin D, Jepsen KJ 2014 Intracortical remodeling parameters are associated with measures of bone robustness: intracortical remodeling and bone robustness. *Anat. Rec.* 297, 1817–1828. (doi:10.1002/ar.22962) 10.1002/ar.22962 PMC4167223 24962664

[15] Padian K, Werning S, Horner JR 2016 A hypothesis of differential secondary bone formation in dinosaurs. *Comptes Rendus Palevol* 15, 40–48. (doi:10.1016/j.crpv.2015.03.002)

[16] Lozupone E, Favia A 1990 The structure of the trabeculae of cancellous bone. 2. Long bones and mastoid. *Calcif. Tissue Int.* 46, 367–372. (doi:10.1007/BF02554966) 2364323 10.1007/BF02554966

[17] Chen H, Senda T, Kubo K 2015 The osteocyte plays multiple roles in bone remodeling and mineral homeostasis. *Med. Mol. Morphol.* 48, 61–68. (doi:10.1007/s00795-015-0099-y) 25791218 10.1007/s00795-015-0099-y

[18] Atkins A *et al.* 2014 Remodeling in bone without osteocytes: Billfish challenge bone structure–function paradigms. *Proc. Natl Acad. Sci. USA* 111, 16 047–16 052. (doi:10.1073/pnas.1412372111) 10.1073/pnas.1412372111 PMC4234560 25331870

[19] Bentolila V, Boyce T, Fyhrie D, Drumb R, Skerry T, Schaffler M 1998 Intracortical remodeling in adult rat long bones after fatigue loading. *Bone* 23, 275–281. (doi:10.1016/S8756-3282(98)00104-5) 9737350 10.1016/s8756-3282(98)00104-5

[20] Currey JD 2002 *Bones: structure and mechanics*. Princeton, NJ: Princeton University Press.

[21] Doube M, Klosowski MM, Wiktorowicz-Conroy AM, Hutchinson JR, Shefelbine SJ 2011 Trabecular bone scales allometrically in mammals and birds. *Proc. R. Soc. B* 278, 3067–3073. (doi:10.1098/rspb.2011.0069) 10.1098/rspb.2011.0069 PMC3158937 21389033

[22] Alexander R, Jayes AS, Maloiy GMO, Wathuta EM 1979 Allometry of the limb bones of mammals from shrews (*Sorex*) to elephant (*Loxodonta*). *J. Zool.* 189, 305–314. (doi:10.1111/j.1469-7998.1979.tb03964.x)

[23] Biewener AA 1983 Allometry of quadrupedal locomotion: the scaling of duty factor, bone curvature and limb orientation to body size. *J. Exp. Biol.* 105, 147–171. 6619724 10.1242/jeb.105.1.147

[24] Christiansen P 1999 Scaling of the limb long bones to body mass in terrestrial mammals. *J. Morphol.* 239, 167–190. (doi:10.1002/(SICI)1097-4687(199902)239:2<167::AID-JMOR5>3.0.CO;2-8) 9951716 10.1002/(SICI)1097-4687(199902)239:2<167::AID-JMOR5>3.0.CO;2-8

[25] Swartz SM, Parker A, Huo C 1998 Theoretical and empirical scaling patterns and topological homology in bone trabeculae. *J. Exp. Biol.* 201, 573–590. 9438832 10.1242/jeb.201.4.573

[26] Ryan TM, Shaw CN 2013 Trabecular bone microstructure scales allometrically in the primate humerus and femur. *Proc. R. Soc. B* 280, 20130172 (doi:10.1098/rspb.2013.0172) 10.1098/rspb.2013.0172 PMC3619467 23486443

[27] Jowsey J 1966 Studies of Haversian systems in man and some animals. *J. Anat.* 100, 857–864. 4961449 PMC1270831

[28] Mishra S, Knothe Tate M 2004 Allometric scaling relationships in microarchitecture of mammalian cortical bone. In *50th Annual Meeting of the Orthopaedic Research Society, San Francisco*, vol. 29, pp. 21–31.

[29] Mishra S 2009 Biomechanical aspects of bone microstructure in vertebrates: potential approach to palaeontological investigations. *J. Biosci.* 34, 799–809. (doi:10.1007/s12038-009-0061-z) 20009272 10.1007/s12038-009-0061-z

[30] Tarach J, Czaja M 1973 Statistical analysis of some size parameters of Haversian systems in femoral, ground transverse sections in man and animals (author’s translation). *Ann. Univ. Mariae. Curie. Sklodowska. Med.* 28, 99–105. 4789864

[31] Warton DI, Wright IJ, Falster DS, Westoby M 2006 Bivariate line-fitting methods for allometry. *Biol. Rev.* 81, 259 (doi:10.1017/S1464793106007007) 16573844 10.1017/S1464793106007007

[32] Felsenstein J 1985 Phylogenies and the comparative method. *Am. Nat.* 125, 1–15. (doi:10.1086/284325)

[33] Pfeiffer S, Crowder C, Harrington L, Brown M 2006 Secondary osteon and Haversian canal dimensions as behavioral indicators. *Am. J. Phys. Anthropol.* 131, 460–468. (doi:10.1002/ajpa.20454) 16685724 10.1002/ajpa.20454

[34] Hidaka S, Matsumoto M, Ohsako S, Toyoshima Y, Nishinakagawa H 1998 A histometrical study on the long bones of raccoon dogs, *Nyctereutes procyonoides* and badgers, *Meles meles*. *J. Vet. Med. Sci.* 60, 323–326. (doi:10.1292/jvms.60.323) 9560780 10.1292/jvms.60.323

[35] Havill LM 2003 Osteon remodeling dynamics in *Macaca mulatta*: normal variation with regard to age, sex, and skeletal maturity. *Calcif. Tissue Int.* 74, 95–102. (doi:10.1007/s00223-003-9038-3) 14973638 10.1007/s00223-003-9038-3

[36] Martiniaková M, Grosskopf B, Omelka R, Vondráková M, Bauerová M 2006 Differences among species in compact bone tissue microstructure of mammalian skeleton: use of a discriminant function analysis for species identification. *J. Forensic Sci.* 51, 1235–1239. (doi:10.1111/j.1556-4029.2006.00260.x) 17199608 10.1111/j.1556-4029.2006.00260.x

[37] Zedda M, Lepore G, Manca P, Chisu V, Farina V 2008 Comparative bone histology of adult horses (*Equus caballus*) and cows (*Bos taurus*). *Anat. Histol. Embryol.* 37, 442–445. (doi:10.1111/j.1439-0264.2008.00878.x) 18671686 10.1111/j.1439-0264.2008.00878.x

[38] Giua S, Farina V, Cacchioli A, Ravanetti F, Carcupino M, Novas MM, Zedda M 2014 Comparative histology of the femur between mouflon (*Ovis aries musimon*) and sheep (*Ovis aries aries*). *J. Biol. Res.—Bollettino della Società Italiana di Biologia Sperimentale* 87, 20144743 (doi:10.4081/jbr.2014.4743)

[39] Brits D, Steyn M, L’Abbé EN 2014 A histomorphological analysis of human and non-human femora. *Int. J. Legal. Med.* 128, 369–377. (doi:10.1007/s00414-013-0854-3) 23604414 10.1007/s00414-013-0854-3

[40] Nganvongpanit K, Siengdee P, Buddhachat K, Brown JL, Klinhom S, Pitakarnnop T, Angkawanish T, Thitaram C 2016 Anatomy, histology and elemental profile of long bones and ribs of the Asian elephant (*Elephas maximus*). *Anat. Sci. Int.* 92, 554–568. (doi:10.1007/s12565-016-0361-y) 27491825 10.1007/s12565-016-0361-y

[41] Quekett JT 1855 *Descriptive and illustrated catalogue of the histological series contained in the museum of the Royal College of Surgeons of England prepared for the microscope: structure of the skeleton of vertebrate animals*, vol. 2 London, UK: Taylor and Francis. PMC5210837 30165008

[42] Jones KE *et al.* 2009 PanTHERIA: a species-level database of life history, ecology, and geography of extant and recently extinct mammals: *Ecological Archives* E090-184. *Ecology* 90, 2648–2648 (doi:10.1890/08-1494.1)

[43] Larramendi A 2015 Proboscideans: shoulder height, body mass and shape. *Acta Palaeontol. Pol.* 61, 537–574. (doi:10.4202/app.00136.2014)

[44] McBee K, Baker RJ 1982 Dasypus novemcinctus. *Mammalian Species* 1–9. (doi:10.2307/3503864)

[45] Swartz SM, Middleton KM 2008 Biomechanics of the bat limb skeleton: scaling, material properties and mechanics. *Cells Tissues Organs* 187, 59–84. (doi:10.1159/000109964) 18160803 10.1159/000109964

[46] Hershkovitz P 1966 *Catalog of living whales*. Washington DC: Smithsonian Institution.

[47] Peters W, Doria G 1876 Descrizione di una nuova specie di Tachyglossus, proveniente dalla Nuova Guinea settentrionale. *Annali del Museo civico di Storia naturale di Genova* IX, 183–187.

[48] Kerbert C 1913 Mitteilungen über Zaglossus. *Bijdragen tot de Dierkunde* 1, 165–184.

[49] Linnaeus C 1806 *Systema naturae*, vol. I London, UK: Lackington, Allen, and Co.

[50] Anderson JF, Hall-Martin A, Russell DA 1985 Long-bone circumference and weight in mammals, birds and dinosaurs. *J. Zool.* 207, 53–61. (doi:10.1111/j.1469-7998.1985.tb04915.x)

[51] Gray JE 1866 *Catalogue of seals and whales in the British Museum*, 2nd edn London, UK: Order of the Trustees.

[52] Hamilton JE 1940 On the history of the elephant seal, *Mirounga leonina* (Linn.). *Proc. Linnean Soc. Lond.* 152, 33–37. (doi:10.1111/j.1095-8312.1940.tb00242.x)

[53] Groves CP 2005 Order Diprotodontia. In *Mammal species of the world: a taxonomic and geographic reference* (eds DE Wilson, DM Reeder), vol. 110, 3rd edn, pp. 43–70. Baltimore, MD: Johns Hopkins University Press.

[54] Falconer H, Walker H 1859 *Descriptive catalogue of the fossil remains of vertebrata from the Sewalik Hills, the Nerbudda, Perim Island, etc. in the Museum of the Asiatic Society of Bengal*. Calcutta, India: Baptist Mission Press.

[55] Shoshani J, Tassy P 2005 Advances in proboscidean taxonomy & classification, anatomy & physiology, and ecology & behavior. *Quat. Int.* 126–128, 5–20. (doi:10.1016/j.quaint.2004.04.011)

[56] Stafford BJ 2005 Order Dermoptera. In *Mammal species of the world: a taxonomic and geographic reference* (eds DE Wilson, DM Reeder), 3rd edn, vol. 110. Baltimore, MD: Johns Hopkins University Press.

[57] Poole WE 1982 Macropus giganteus. *Mammalian Species* 1–8. (doi:10.2307/3504005)

[58] Quekett JT 1852 Practical treatise on the use of the microscope. In *Illustrated standard scientific works*, vol. 6, 2nd edn. London, UK: H. Bailliere.

[59] Straehl FR, Scheyer TM, Forasiepi AM, MacPhee RD, Sánchez-Villagra MR 2013 Evolutionary patterns of bone histology and bone compactness in Xenarthran mammal long bones. *PLoS ONE* 8, e69275 (doi:10.1371/journal.pone.0069275) 23874932 10.1371/journal.pone.0069275 PMC3706384

[60] Houssaye A 2009 ‘Pachyostosis’ in aquatic amniotes: a review. *Integr. Zool.* 4, 325–340. (doi:10.1111/j.1749-4877.2009.00146.x) 21392306 10.1111/j.1749-4877.2009.00146.x

[61] Preibisch S, Saalfeld S, Tomancak P 2009 Globally optimal stitching of tiled 3D microscopic image acquisitions. *Bioinformatics* 25, 1463–1465. (doi:10.1093/bioinformatics/btp184) 19346324 10.1093/bioinformatics/btp184 PMC2682522

[62] Schindelin J *et al.* 2012 Fiji: an open-source platform for biological-image analysis. *Nat. Methods* 9, 676–682. (doi:10.1038/nmeth.2019) 22743772 10.1038/nmeth.2019 PMC3855844

[63] Robling AG, Stout SD 1999 Morphology of the drifting osteon. *Cells Tissues Organs* 164, 192–204. (doi:10.1159/000016659) 10436327 10.1159/000016659

[64] R Core Team. R: A language and environment for statistical computing. Vienna, Austria: R Foundation for Statistical Computing. (https://www.R-project.org/)

[65] Warton DI, Duursma RA, Falster DS, Taskinen S 2012 smatr 3- an R package for estimation and inference about allometric lines. *Methods Ecol. Evol.* 3, 257–259. (doi:10.1111/j.2041-210X.2011.00153.x)

[66] Schmidt-Nielsen K 1975 Scaling in biology: the consequences of size. *J. Exp. Zool.* 194, 287–307. (doi:10.1002/jez.1401940120) 811757 10.1002/jez.1401940120

[67] Paradis E, Claude J, Strimmer K 2004 APE: analyses of phylogenetics and evolution in R language. *Bioinformatics* 20, 289–290. (doi:10.1093/bioinformatics/btg412) 14734327 10.1093/bioinformatics/btg412

[68] Bininda-Emonds ORP *et al.* 2007 The delayed rise of present-day mammals. *Nature* 446, 507–512. (doi:10.1038/nature05634) 17392779 10.1038/nature05634

[69] Rohland N, Malaspinas AS, Pollack JL, Slatkin M, Matheus P, Hofreiter M 2007 Proboscidean mitogenomics: chronology and mode of elephant evolution using mastodon as outgroup. *PLoS Biol.* 5, e207 (doi:10.1371/journal.pbio.0050207) 17676977 10.1371/journal.pbio.0050207 PMC1925134

[70] Felder A Submitted Alessandrofelder/Pangolin. (doi:10.5281/zenodo.556370)

[71] Felder A Submitted. Alessandrofelder/Wallaby. (doi:10.5281/zenodo.556370doi:10.5281/zenodo.495368)

[72] Qiu S, Rao DS, Palnitkar S, Parfitt AM 2010 Dependence of bone yield (volume of bone formed per unit of cement surface area) on resorption cavity size during osteonal remodeling in human rib: implications for osteoblast function and the pathogenesis of age-related bone loss. *J. Bone. Miner. Res.* 25, 423–430. (doi:10.1359/jbmr.091003) 19821766 10.1359/jbmr.091003 PMC3153391

[73] Skedros JG *et al.* 2013 Scaling of Haversian canal surface area to secondary osteon bone volume in ribs and limb bones. *Am. J. Phys. Anthropol.* 151, 230–244. (doi:10.1002/ajpa.22270) 23633395 10.1002/ajpa.22270

[74] Wachter NJ, Krischak GD, Mentzel M, Sarkar MR, Ebinger T, Kinzl L, Claes L, Augat P 2002 Correlation of bone mineral density with strength and microstructural parameters of cortical bone in vitro. *Bone* 31, 90–95. (doi:10.1016/S8756-3282(02)00779-2) 12110418 10.1016/s8756-3282(02)00779-2

[75] Savage VM, Allen AP, Brown JH, Gillooly JF, Herman AB, Woodruff WH, West GB 2007 Scaling of number, size, and metabolic rate of cells with body size in mammals. *Proc. Natl Acad. Sci. USA* 104, 4718–4723. (doi:10.1073/pnas.0611235104) 17360590 10.1073/pnas.0611235104 PMC1838666

[76] Gregory TR 2000 Nucleotypic effects without nuclei: genome size and erythrocyte size in mammals. *Genome* 43, 895–901. (doi:10.1139/g00-069) 11081981 10.1139/g00-069

[77] Currey JD, Shahar R 2013 Cavities in the compact bone in tetrapods and fish and their effect on mechanical properties. *J. Struct. Biol.* 183, 107–122. (doi:10.1016/j.jsb.2013.04.012) 23664869 10.1016/j.jsb.2013.04.012

[78] Piekarski K 1970 Fracture of bone. *J. Appl. Phys.* 41, 215–223. (doi:10.1063/1.1658323)

[79] Moyle DD, Bowden RW 1984 Fracture of human femoral bone. *J. Biomech.* 17, 203–213. (doi:10.1016/0021-9290(84)90011-3) 6736057 10.1016/0021-9290(84)90011-3

[80] Pope MH, Murphy MC 1974 Fracture energy of bone in a shear mode. *Med. Biol. Eng.* 12, 763–767. (doi:10.1007/BF02477442) 4467003 10.1007/BF02477442

[81] Corondan G, Haworth WL 1986 A fractographic study of human long bone. *J. Biomech.* 19, 207–218. (doi:10.1016/0021-9290(86)90153-3) 3700433 10.1016/0021-9290(86)90153-3

[82] Bernhard A *et al.* 2013 Micro-morphological properties of osteons reveal changes in cortical bone stability during aging, osteoporosis, and bisphosphonate treatment in women. *Osteoporos. Int.* 24, 2671–2680. (doi:10.1007/s00198-013-2374-x) 23632826 10.1007/s00198-013-2374-x

[83] Brianza SZM, D’Amelio P, Pugno N, Zini E, Zatelli A, Pluviano F, Cabiale K, Galloni M, Isaia GC 2011 Microdamage accumulation changes according to animal mass: an intraspecies investigation. *Calcif. Tissue Int.* 88, 409–415. (doi:10.1007/s00223-011-9470-8) 21331568 10.1007/s00223-011-9470-8

[84] Le JL, Bažant ZP 2011 Unified nano-mechanics based probabilistic theory of quasibrittle and brittle structures: II. Fatigue crack growth, lifetime and scaling. *J. Mech. Phys. Solids.* 59, 1322–1337. (doi:10.1016/j.jmps.2011.03.007)

[85] Kim KT, Bažant ZP, Yu Q 2013 Non-uniqueness of cohesive-crack stress-separation law of human and bovine bones and remedy by size effect tests. *Int. J. Fract.* 181, 67–81. (doi:10.1007/s10704-013-9821-8)

[86] Biewener AA 1989 Scaling body support in mammals: limb posture and muscle mechanics. *Science* 245, 45–48. (doi:10.1126/science.2740914) 2740914 10.1126/science.2740914

[87] Campione NE, Evans DC 2012 A universal scaling relationship between body mass and proximal limb bone dimensions in quadrupedal terrestrial tetrapods. *BMC Biol.* 10, 60 (doi:10.1186/1741-7007-10-60) 22781121 10.1186/1741-7007-10-60 PMC3403949

[88] Houssaye A, Waskow K, Hayashi S, Cornette R, Lee AH, Hutchinson JR 2016 Biomechanical evolution of solid bones in large animals: a microanatomical investigation. *Biol. J. Linnean Soc.* 117, 350–371. (doi:10.1111/bij.12660)

[89] Mishra S, Knothe Tate ML 2003 Effect of lacunocanalicular architecture on hydraulic conductance in bone tissue: implications for bone health and evolution. *Anat. Rec.* 273A, 752–762. (doi:10.1002/ar.a.10079) 10.1002/ar.a.10079 12845711

[90] Kamioka et al 2012 Microscale fluid flow analysis in a human osteocyte canaliculus using a realistic high-resolution image-based three-dimensional model. Integr. Biol. 4, 1198–1206. (doi:10.1039/c2ib20092a) 10.1039/c2ib20092a 22858651

[91] Bromage TG *et al.* 2009 Lamellar bone is an incremental tissue reconciling enamel rhythms, body size, and organismal life history. *Calcif. Tissue Int.* 84, 388–404. (doi:10.1007/s00223-009-9221-2) 19234658 10.1007/s00223-009-9221-2

[92] Ritvo H 1990 *The animal estate: the English and other creatures in the Victorian Age*. Cambridge, MA: Harvard University Press.

[93] Kleiman DG (ed.). 1996 *Wild mammals in captivity: principles and techniques*. Chicago, IL: University of Chicago Press.

[94] Britz HM, Thomas CDL, Clement JG, Cooper DM 2009 The relation of femoral osteon geometry to age, sex, height and weight. *Bone* 45, 77–83. (doi:10.1016/j.bone.2009.03.654) 19303955 10.1016/j.bone.2009.03.654

[95] Dominguez VM, Agnew AM 2016 Examination of factors potentially influencing osteon size in the human rib. *Anat. Rec.* 299, 313–324. (doi:10.1002/ar.23305) 10.1002/ar.23305 26692499

[96] Lieberman DE, Polk JD, Demes B 2004 Predicting long bone loading from cross-sectional geometry. *Am. J. Phys. Anthropol.* 123, 156–171. (doi:10.1002/ajpa.10316) 14730649 10.1002/ajpa.10316

[97] Skedros JG, Clark GC, Sorenson SM, Taylor KW, Qiu S 2011 Analysis of the effect of osteon diameter on the potential relationship of osteocyte lacuna density and osteon wall thickness. *Anat. Rec.: Adv. Integr. Anat. Evol. Biol.* 294, 1472–1485. (doi:10.1002/ar.21452) 10.1002/ar.21452 PMC3277654 21809466

[98] Currey J 2003 The many adaptations of bone. *J. Biomech.* 36, 1487–1495. (doi:10.1016/S0021-9290(03)00124-6) 14499297 10.1016/s0021-9290(03)00124-6

[99] Harrison KD, Cooper DML 2015 Modalities for visualization of cortical bone remodeling: the past, present, and future. *Front. Endocrinol. (Lausanne)* 6, 122 (doi:10.3389/fendo.2015.00122) 26322017 10.3389/fendo.2015.00122 PMC4531299

[100] Felder A, Phillips C, Cornish H, Cooke M, Hutchinson JR, Doube M 2017 Data from: Secondary osteons scale allometrically in the mammalian humerus and femur. Dryad Digital Repository (doi:10.5061/dryad.637r1) 10.1098/rsos.170431 PMC5717626 29291052

